# The Alpha-1A Adrenergic Receptor in the Rabbit Heart

**DOI:** 10.1371/journal.pone.0155238

**Published:** 2016-06-03

**Authors:** R. Croft Thomas, Patrick M. Cowley, Abhishek Singh, Bat-Erdene Myagmar, Philip M. Swigart, Anthony J. Baker, Paul C. Simpson

**Affiliations:** 1 Department of Medicine, Cardiology Division, and Research Service, VA Medical Center, San Francisco, CA, United States of America; 2 Department of Medicine and Cardiovascular Research Institute, University of California San Francisco, San Francisco, CA, United States of America; Temple University, UNITED STATES

## Abstract

The alpha-1A-adrenergic receptor (AR) subtype is associated with cardioprotective signaling in the mouse and human heart. The rabbit is useful for cardiac disease modeling, but data on the alpha-1A in the rabbit heart are limited. Our objective was to test for expression and function of the alpha-1A in rabbit heart. By quantitative real-time reverse transcription PCR (qPCR) on mRNA from ventricular myocardium of adult male New Zealand White rabbits, the alpha-1B was 99% of total alpha-1-AR mRNA, with <1% alpha-1A and alpha-1D, whereas alpha-1A mRNA was over 50% of total in brain and liver. Saturation radioligand binding identified ~4 fmol total alpha-1-ARs per mg myocardial protein, with 17% alpha-1A by competition with the selective antagonist 5-methylurapidil. The alpha-1D was not detected by competition with BMY-7378, indicating that 83% of alpha-1-ARs were alpha-1B. In isolated left ventricle and right ventricle, the selective alpha-1A agonist A61603 stimulated a negative inotropic effect, versus a positive inotropic effect with the nonselective alpha-1-agonist phenylephrine and the beta-agonist isoproterenol. Blood pressure assay in conscious rabbits using an indwelling aortic telemeter showed that A61603 by bolus intravenous dosing increased mean arterial pressure by 20 mm Hg at 0.14 μg/kg, 10-fold lower than norepinephrine, and chronic A61603 infusion by iPRECIO programmable micro Infusion pump did not increase BP at 22 μg/kg/d. A myocardial slice model useful in human myocardium and an anthracycline cardiotoxicity model useful in mouse were both problematic in rabbit. We conclude that alpha-1A mRNA is very low in rabbit heart, but the receptor is present by binding and mediates a negative inotropic response. Expression and function of the alpha-1A in rabbit heart differ from mouse and human, but the vasopressor response is similar to mouse.

## Introduction

Evidence for an important role for α1-adrenergic receptors (ARs) in cardiac myocyte physiology has accumulated over several decades (reviewed in [1,2,3]), even though these receptors are much less abundant than β-ARs. Among the 3 α1-AR subtypes, A, B, and D, the α1A is of special interest, since this subtype can mediate adaptive and protective effects in mouse models [4,5,6,7], and can stimulate protective ERK signaling and contraction in human myocardium [8].

To test signaling discovered in rodents, the rabbit offers a medium size animal with multiple cardiac disease models. The rabbit has the advantage over rodents of greater similarity to human myocardium, in terms of electrophysiology, calcium handling, and myosin isoform expression [9], and several studies show α1-AR-mediated cardiac protection against ischemia-reperfusion injury in rabbits (reviewed in [1]).

However, whether the α1A subtype is important in α1-AR effects in the rabbit heart is uncertain. One group finds negligible α1A mRNA and binding in rabbit heart [10,11], whereas others report that the α1A is 12–27% of total α1-AR binding [12,13]. Whether the α1A is functional in rabbit myocardium has been assessed mainly by antagonism of phenylephrine (PE)-induced positive inotropic effects by antagonists of uncertain selectivity, and the results are confusing [12,13,14,15].

We wished to test whether α1A cardioprotective and contractile effects discovered in the mouse [4,5,6,7] were seen in the rabbit. Here we measured α1-AR subtype mRNAs and binding in rabbit myocardium, and tested myocardial inotropic effects and blood pressure (BP) responses to α1-agonists. We find that α1A expression and function in rabbit heart differs from mouse and human, whereas the vasopressor response is similar to mouse. We also note unsuccessful application to rabbit of a myocardial slice model useful in human myocardium and an anthracycline cardiotoxicity model useful in the mouse.

## Material and Methods

### Rabbits

Adult male New Zealand White Rabbits weighing 3.25–4.0 kg were from Western Oregon Rabbit Company (Philomath, OR). Upon delivery, rabbits were singly housed in the Veterinary Medical Unit in stainless steel, ventilated rabbit racks and allowed to assimilate for one week. Room temperature was 62–72°F, with humidity 30–70%, and the light cycle was 12 h (6 am to 6 pm). Rabbits were fed 8 oz daily Teklad Global High Fiber Rabbit Diet #2031, plus occasional alfalfa and vegetables (4 oz). Surgery was done in a dedicated suite. Euthanasia was by cardiectomy under deep anesthesia with isoflurane. All procedures were reviewed and approved by the San Francisco VA Medical Center Animal Care and Use Committee.

### RNA preparation

Tissue was homogenized in TRIzol reagent (ThermoFisher Scientific #15596–018), using a rotor-stator homogenizer (Polytron) at speed 7 out of 10 at 4°C. RNA was extracted with chloroform (Fisher Scientific #C606-1), and the aqueous phase was purified on Qiagen Mini-Prep columns (RNeasy Mini Kit #74106). Samples were treated with DNase (Turbo DNAfree, Ambion #AM1907), and quantified using spectrophotometry (BioRad SmartSpec 3000). A260/280 ratios were at least 1.9. Selected RNA samples were analyzed to confirm the absence of significant degradation (Agilent 2100 BioAnalyzer).

### Quantitative real-time reverse transcription polymerase chain reaction (qPCR)

Primer3 (v0.4.0) and NCBI BLAST were used to design primer pairs for each target and reference gene, based on *Oryctolagus Cuniculus* sequence. α1-AR subtype primers spanned the 25 kb intron at the end of the 6^th^ transmembrane domain, to avoid amplification of contaminating genomic DNA.

ADRA1A (adrenoceptor alpha 1A) NM_001082380.1

Forward AGGCTCCTCAAGTTTTCCCG

Reverse AGTTTCCGGGGGCTTGAAAT

ADRA1B (adrenoceptor alpha 1B) NM_001082062.1

Forward TCTTGTGCTGGCTTCCCTTC

Reverse CGCTTGAACTCCTTGCTGGA

ADRA1D (adrenoceptor alpha 1D) NM_001082678.1

Forward CTCCAGCCTGTCGCACAAGA

Reverse AAATGTCAGTCTCGCGGAGG

GAPDH (glyceraldehyde-3-phosphate dehydrogenase) NM_001082253.1

Forward AGACACGATGGTGAAGGTCG

Reverse TGCCGTGGGTGGAATCATAC

HPRT1 (hypoxanthine phosphoribosyltransferase 1) NM_001105671.1

Forward CGTCGAGGACTTGGAAAGGG

Reverse TTGAGCACACAGAGGGCTAC

EEF1A1 (eukaryotic translation elongation factor 1 alpha 1) NM_001082339.1

Forward GGACTGCATCCTTCCACCAA

Reverse GGGACAGTGCCAATACCACC

For qPCR, 1 μg of RNA was reverse transcribed using Transcriptor First Strand cDNA Synthesis Kit (Roche #04 897 030 001) with both random hexamers and oligo-dT. qPCR reactions contained 5% of the cDNA product, primers at 125 nM per reaction, and SYBR Green Master (Roche) with ROX reference dye. All reactions were performed in triplicate in an ABI PRISM 7900HT Sequence Detection System. Data were analyzed with SDS software version 2.3 (Applied Biosystems).

Relative quantitation of PCR products used the ΔΔCt method [16]. Values for each mRNA are arbitrary units (AU) relative to three reference genes, GAPDH, HPRT1, and EEF1A1, for improved accuracy [17], as AU = 2^-ΔΔCT^ x 1000, where ΔΔCT = [(mean target gene C_T_)—(mean reference genes C_T_)].

### Radioligand binding

Approximately 200 mg wet weight of tissue was homogenized (5 mM Tris-HCl, 5 mM EDTA, 250 M Sucrose pH 7.4 plus 0.1 mM PMSF), and centrifuged at 100,000 x *g* for 1 h. The pellet was resuspended in homogenization buffer and centrifuged again. The resulting final membrane pellet was resuspended in assay buffer (50 mM Tris pH 7.4, 1 mM EDTA), and used for saturation and competition radioligand binding.

α1-AR saturation binding was at 30°C for 60 min with 50–200 μg membrane protein per tube (~2.5 mg tissue), in triplicate with 6 concentrations (0.04–1.2 nM) of ^3^H-prazosin (85 Ci/mmol, Perkin Elmer #NET-823), or 6 concentrations (10–800 pM) ^125^I-HEAT (2-[β-(4-hydroxyphenyl)-ethyl-aminomethyl] tetralone) (2200 Ci/mmol, Perkin Elmer #NEX182100UC). Phentolamine (10 μM) (Sigma #P-131) defined non-specific binding [18].

The subtype proteins were quantified by competition binding. Binding of ^3^H-prazosin (0.5 nM) or ^125^I-HEAT (50 pM) was competed with 22 concentrations (0.05 nM—500 μM) in duplicate of BMY-7378 (Sigma Aldrich #B134), an α1D-selective antagonist [19,20], or 5-methylurapidil (5MU) (Sigma-Aldrich #U101), an α1A-selective antagonist [21]. Binding data were analyzed using GraphPad Prism v5.0 (GraphPad Software Inc., San Diego, CA). Subtype percents were calculated from fitting competition curves.

### Myocardial contraction

Linear unbranched trabeculae from the left ventricle (LV) and right ventricle (RV) were dissected in modified Krebs-Henseleit solution containing (in mM) NaCl, 137; KCl, 10; MgSO4, 1.2; NaH2PO4, 1.2; glucose, 10; NaHCO3, 20; CaCl2, 0.2; and 2,3-butanedione monoxime, 30; and gassed with 95% O2/5% CO2 to give a pH of 7.4 at 22°C. Trabeculae remained in this solution until use. Two trabeculae per heart, one from each ventricle, were studied consecutively, with the order of use alternated between experiments. Trabeculae were placed in a muscle chamber (3 x 3 x 15 mm) and mounted between a force transducer (AE-801, Kronex, Oakland, CA), and a micromanipulator using stainless steel pins.

Trabeculae were superfused at 5 ml/min. for 1 h at room temperature in Krebs-Henseleit solution containing 5 mM KCL and no 2,3,-butanedione monoxime. CaCl2 was gradually increased over 30 min. to 1.5 mM. The solution temperature was increased to 36.5°C. Trabeculae were electrically stimulated at 1.5 Hz using platinum wire electrodes, and the muscle length was increased to maximize the contraction force.

Cardiac α1-adrenergic receptors were stimulated by addition of a maximal dose of the subtype-nonselective agonist phenylephrine (PE) (Sigma #P-6126) (10 μM), or a maximal dose of the α1A-subtype-selective agonist A61603 (*N*-[5-(4,5-dihydro-1*H*-imidazol-2-yl)-2-hydroxy-5,6,7,8-tetrahydronaphthalen-1-yl]methanesulfonamide hydrobromide) (100 nM) (Tocris Pharmaceuticals #1052). The order was alternated between experiments. Acute inotropic responses to α1-adrenergic receptor agonists were assessed when contraction force stabilized, typically ~20 min after addition. After each agonist treatment, agonist was then removed by perfusion for 20 min. with drug-free solution.

Cardiac β-adrenergic receptors were stimulated by addition of a maximal dose (1μM) of subtype-nonselective agonist L-isoproterenol HCl (ISO) (Sigma #I6504). The inotropic response to ISO was measured 150–200 seconds after addition, when the contraction force was maximal.

### Telemetry for blood pressure (BP) and heart rate (HR)

BP and HR were measured in awake rabbits using implanted telemeters. Pressure waveforms were acquired at 500 Hz and reduced to 5–20 s means. All telemetry equipment was purchased from Data Sciences International (DSI) (St. Paul, MN) including telemeters (model TL11-M2-D70-PCT 25 cm catheter, #270-0093-816), receivers (model RPC-1, #272-6001-001), a data processing device (model Data Exchange Matrix, #271-0117-001), an ambient pressure reference monitor (model APR-1, #275-0020-001), and Dataquest A.R.T software with Dell computer (model Dataquest A.R.T., #271-0147-CFG). Data were analyzed in Excel (Microsoft), and graphs and statistics used Prism 6.0 (GraphPad).

For telemeter implantation, rabbits were sedated with buprenorphine 0.3 mg/kg subcutaneous and combined ketamine 25 mg/kg and xylazine 3 mg/kg intramuscular; then anesthetized with inhaled isoflurane 2–5%; and given 100% oxygen through an endotracheal tube (cuffed, size 2). SpO2, HR, and temperature (37°C) were monitored. The telemeter was threaded into the abdominal aorta through the right femoral artery, which was permanently ligated, and the transmitter was implanted in the right flank. Two ECG leads were placed subcutaneously over the rib cage. Carprofen 4 mg/kg subcutaneous was given for post-operative pain, and enrofloxacin 5 mg/kg subcutaneous was given for antibiotic prophylaxis. Rabbits rested for 7 d after surgery.

### Acute intravenous (IV) dose-response studies

Awake, non-sedated rabbits were gently restrained (Tecniplast Rabbit Restraint box). A 20 gauge IV was inserted into an ear vein, and the rabbits recovered for at least 30 min and until BP and HR normalized. Vehicle and escalating doses of the nonselective AR agonist norepinephrine (Sigma #A0937) and the α1A-AR specific agonist A61603 were infused in 3 ml saline, with at least 5 min between doses for BP and HR to return to baseline. Values were captured every 5 sec; baseline was taken as the 1 min average prior to dosing, and peak was the maximum 10 sec value within 1 min of infusion.

### Chronic drug infusion by iPRECIO

Increasing doses of A61603 were infused using an iPRECIO microinfusion pump (Primetech, Tokyo, Japan). Following anesthesia as above for telemeter implantation, the iPRECIO pump was placed on the back between the scapulae, and a catheter was tunneled and inserted into the jugular vein, which was permanently ligated. After 4 d recovery, increasing doses of A61603 were infused while BP was measured in the awake, non-restrained rabbit by the indwelling telemeter.

### Myocardial slices

An approach to make thin myocardial slices from the rabbit LV followed a protocol we developed for human LV [8], with numerous modifications in an attempt to obtain viable, relaxed slices. Deep anesthesia was obtained with isoflurane 5%; the heart with a maximum length of aorta was removed quickly, and submerged in ice-cold cardioplegia containing (in mM) NaCl 110; KCl 16; CaCl2 1.2; NaHCO3 10; MgCl2 16. The aorta was cannulated and 100 ml of ice-cold cardioplegia was perfused antegrade into the coronary arteries, resulting in a relaxed state. Five or 8 mm diameter cores from the LV free wall or septum were generated using a coring press (Alabama Research and Development, MD5000/53000, Munford, AL) with a cylindrical coring tool (Alabama Research and Development, MP0144 for 8 mm or MP0143 for 5 mm).

Cores were embedded using a tissue embedding unit (Alabama Research and Development, MD2299) in 1.25% to 2% low melting temperature agarose (Agarose II, Amresco 0815) in several different slicing buffers containing (in mM) NaCl 110–130; KCl 15–16; HEPES 0–10; NaHCO3 4.2–10; Na2HPO4 0–0.3; MgSO4-7H2O 0–0.5 or MgCl2 0–16; glucose 0–5.6; CaCl2 0–1.3; and 2,3-butanedione monoxime (BDM, Sigma-Aldrich B0753) 0–30; pH 7.2). Alternately, cores were used directly for slicing without embedding. The core was oriented in the Krumdieck tissue slicer (Alabama Research and Development, MD4000) with the endocardial surface toward the blade, such that the cutting plane was parallel to the myocyte long axis; a weight maintained downward pressure. Slice thickness was set at 150–350 μm. The instrument passed the core repeatedly and automatically across a replaceable stainless steel blade, while immersed in 4°C, sterile slicing buffer. Circulating buffer floated the resultant slices into a glass trap and a collecting tray. If resulting slices were relaxed, then the protocol was planned as for human slices, to add additional CaCl2 at 10 min intervals to increase calcium concentration gradually to 25, 50, 100, 200, 400, 700, and 1000 μM.

### Anthracycline treatment

Rabbits were given daunorubicin (BioVision #1524–1) 3 mg/kg, doxorubicin (Tocris #2252, lot #3B/137005) 1.5 mg/kg, or vehicle for 10–12 weekly IV doses through an ear vein. At the same time, under anesthesia with buprenorphine 0.03 mg/kg and isoflurane 5% for induction and 2.5% for maintenance, echocardiography to measure cardiac function was done with an Acuson Sequoia C256, and cardiac troponin I was quantified as an index of myocardial damage, using a drop of blood from an ear artery in an iSTAT Cardiac Troponin I cartridge, a two-site enzyme-linked immunosorbant assay (Abaxis #600–9009).

### Statistics

Results are mean ± SE. Radioligand binding curves were fit and significant differences were tested in GraphPad Prism v5.0d. A 1-sample t test was used for the contraction data.

## Results

### The α1B is the dominant α1-AR subtype mRNA in rabbit 267 ventricle

**Fig 1:**
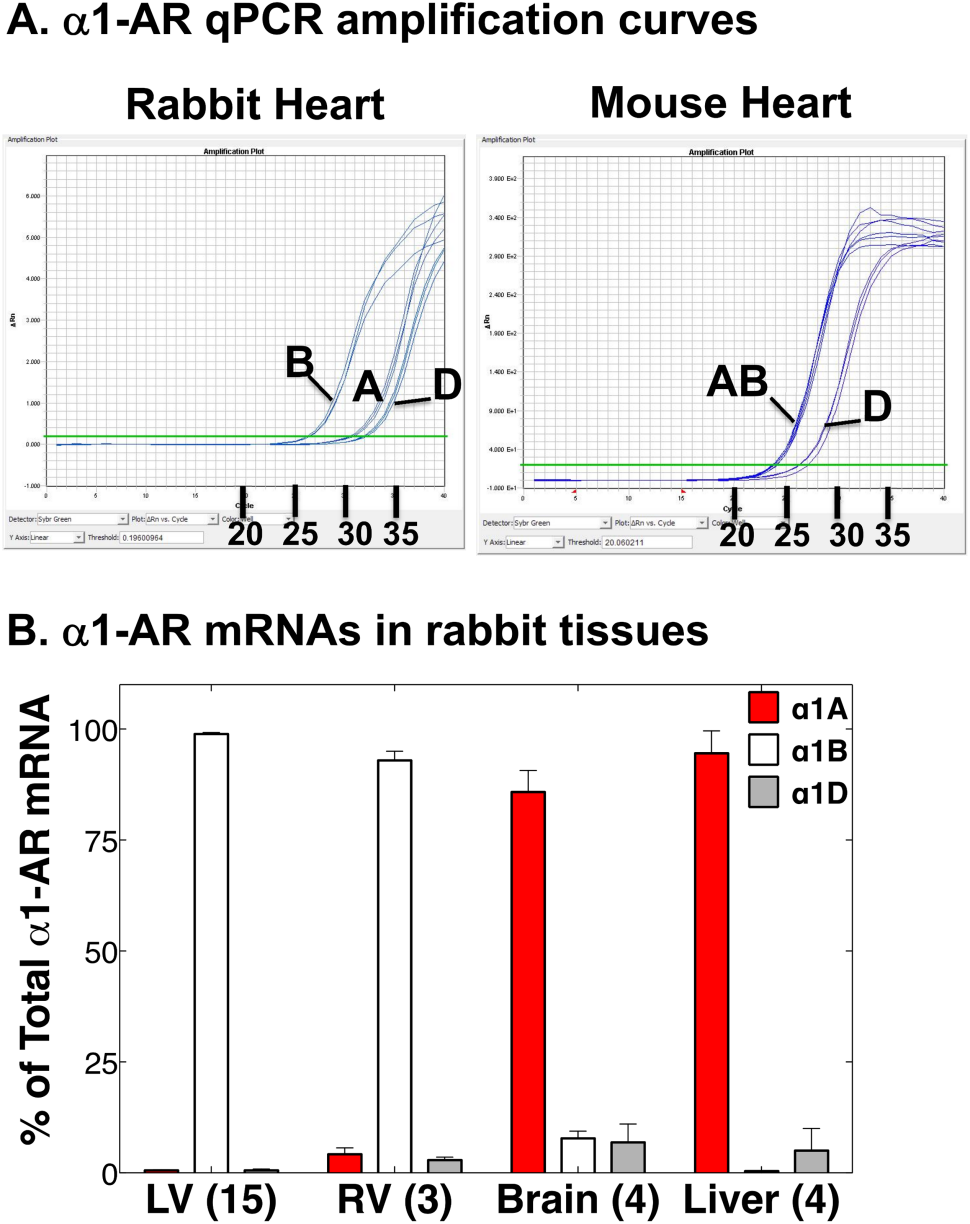
α1A, B, and D subtype mRNAs in the rabbit heart. RNA from the indicated tissues was used in qPCR for the 3 α1-AR subtype mRNAs. (**A**) Original PCR amplification curves for mRNA from a rabbit heart (left), with mouse heart for comparison (right), showing predominant α1B in rabbit with much less α1A and α1D. Number of cycles is indicated on the X axis. (**B**) Group data showing % of total α1-AR mRNA for each α1-AR subtype in rabbit LV, RV, brain, and liver. Values are mean ± SE for the number of animals indicated. α1A mRNA is a very low fraction of total in LV and RV, in contrast with brain and liver as positive controls for the primers and protocol. α1A and α1D mRNAs are somewhat greater in RV than LV, but this might be due to somewhat lower levels of the housekeeping genes in RV.

We quantified the relative levels of α1A, α1B, and α1D mRNAs in rabbit ventricle, using qPCR. As described before for human myocardium [22], the primers were designed on rabbit sequence to cross the large intron between the 2 coding exons, to avoid spurious contamination by genomic DNA. Fig 1A illustrates qPCR amplification curves. The rabbit myocardium curve shows that the α1B mRNA is much more abundant than the α1A or α1D, whereas the mouse myocardium curve for comparison shows that the α1A and α1B are equally abundant. α1B mRNA was >99% of total α1-AR mRNA in the left ventricle (LV) myocardium, and there were trace amounts of α1A and α1D (Fig 1B). α1A and α1D mRNAs were slightly higher in the right ventricle (RV) (Fig 1B). In contrast, and as a control for the primers and protocol, Fig 1B also shows that the α1A was the predominant α1-AR mRNA in brain (85%) and liver (95%), known to have abundant α1A mRNA [10,11].

### The α1A is detectable but low by radioligand binding in rabbit ventricle

We did radioligand binding to test if the α1A was present at the protein level. We could not use α1-AR antibodies, since we find that these antibodies are nonspecific [23]. We used a “total” membrane preparation for binding, which does not discard any low speed pellets. This approach reduced receptor density normalized to protein, but did not discard any receptor binding activity.

**Fig 2:**
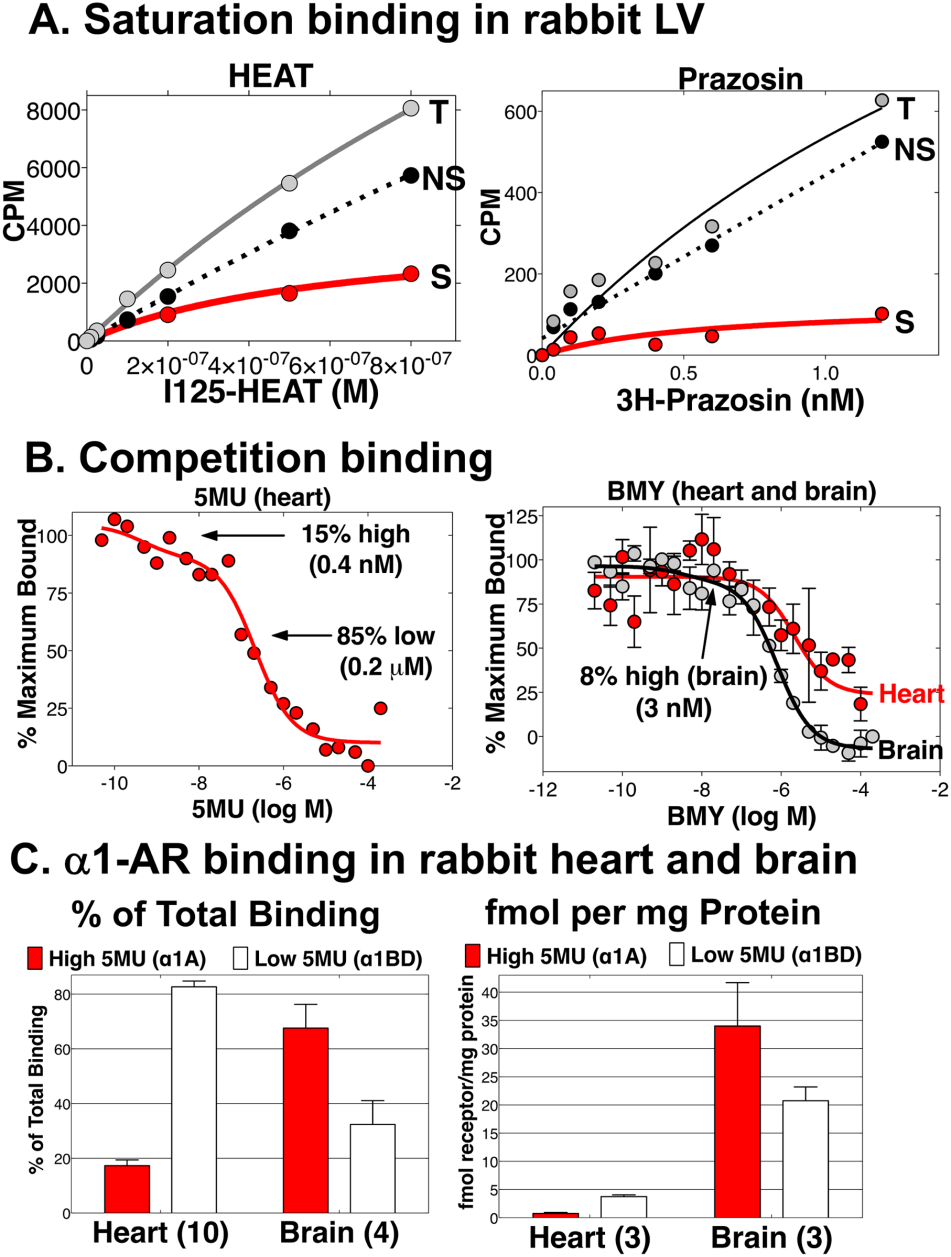
α1AR radioligand binding in the rabbit heart. Total ventricle or brain membranes were prepared and used in binding assays. (**A**) Original saturation binding curves with 200 μg heart membrane protein; **left**, binding with ^125^I-HEAT; **right**, binding with ^3^H-prazosin; T = total binding, NS = nonspecific, S = specific; **(B) left**, competition for ^125^I-HEAT binding in heart with 5-methylurapidil (5MU), an antagonist with high α1A affinity; the 2-site curve shows 15% high affinity binding; **right**, competition in heart, and in brain as a positive control, for ^125^I-HEAT binding with BMY-7378 (BMY), an antagonist with high α1D affinity, shows 8% high affinity binding in brain and none in heart (n = 3). (**C**) Group data showing, **left**, % of total binding with high 5MU affinity (α1A) and low 5MU affinity (α1BD) in heart and brain; and **right**, absolute fmol/mg protein (% x total fmol), from experiments with saturation and competition done in the same preparations.

Fig 2A and 2B show original saturation and competition binding curves in rabbit myocardium. Saturation binding with ^3^H-prazosin or ^125^I-HEAT detected a population of α1-ARs (Fig 2A). The fraction of α1A was estimated by competition with 5MU, since 5MU identifies the same number of α1A-ARs as is identified by α1A knockout [21]. Competition with 5MU for ^3^H-prazosin or ^125^I-HEAT binding produced 2-site curves, with high and low affinity components, as illustrated in Fig 2B left.

In heart, there were 4.7 ± 0.8 fmol total α1-ARs per mg protein (n = 7), with specific binding 34 ± 4% of total at the ligand Kd. There were 17 ± 2% sites with high 5MU affinity (n = 10, log IC50–8.9 ± 0.3), representing the α1A; and 83% ± 2% sites with low affinity (log IC50–5.5 ± 0.4), representing the α1B and/or α1D. In brain for comparison, total α1-ARs were 46 ± 9 fmol per mg protein (n = 4), with specific binding 81 ± 2% of total at the ligand Kd. There were 68 ± 9% sites with high 5MU affinity (n = 4), representing the α1A.

Competition with BMY-7378, an antagonist with high α1D affinity, identified whether the sites with low 5MU affinity were α1B and/or α1D. BMY-7378 competition in heart membranes produced a 1-site curve with a single low affinity site (log IC50–5.67), whereas competition in brain produced a 2-site curve with 8% high affinity sites (log IC50–8.52) and 92% low affinity (log IC50–6.10) (Fig 2B right).

Taken together, these data showed that myocardium had 17% α1A, 83% α1B, and no α1D. In comparison, brain had 68% α1A, 24% α1B, and 8% α1D.

Fig 2C displays the α1A binding data in heart and brain, as percent of total α1-AR binding (left), and as fmol per mg protein (right). To calculate fmol α1A per mg protein we multiplied the percent of sites with high affinity for 5MU by the total α1-AR binding, using saturation and competition analyses done on the same membrane preparations. By this method, the α1A was 0.8 ± 0.2 fmol per mg protein in heart (n = 3), versus 34 ± 8 in brain (n = 3).

### The α1A mediates a negative inotropic effect in rabbit myocardium

To test whether the low levels of the α1A identified in rabbit myocardium were functional, we studied inotropic responses in vitro. Trabeculae from the RV and LV were paced at 37°C and treated with A61603. A61603 is a highly selective α1A agonist [24], that requires the α1A for activity, as shown in experiments with the α1A knockout [21,25]).

**Fig 3:**
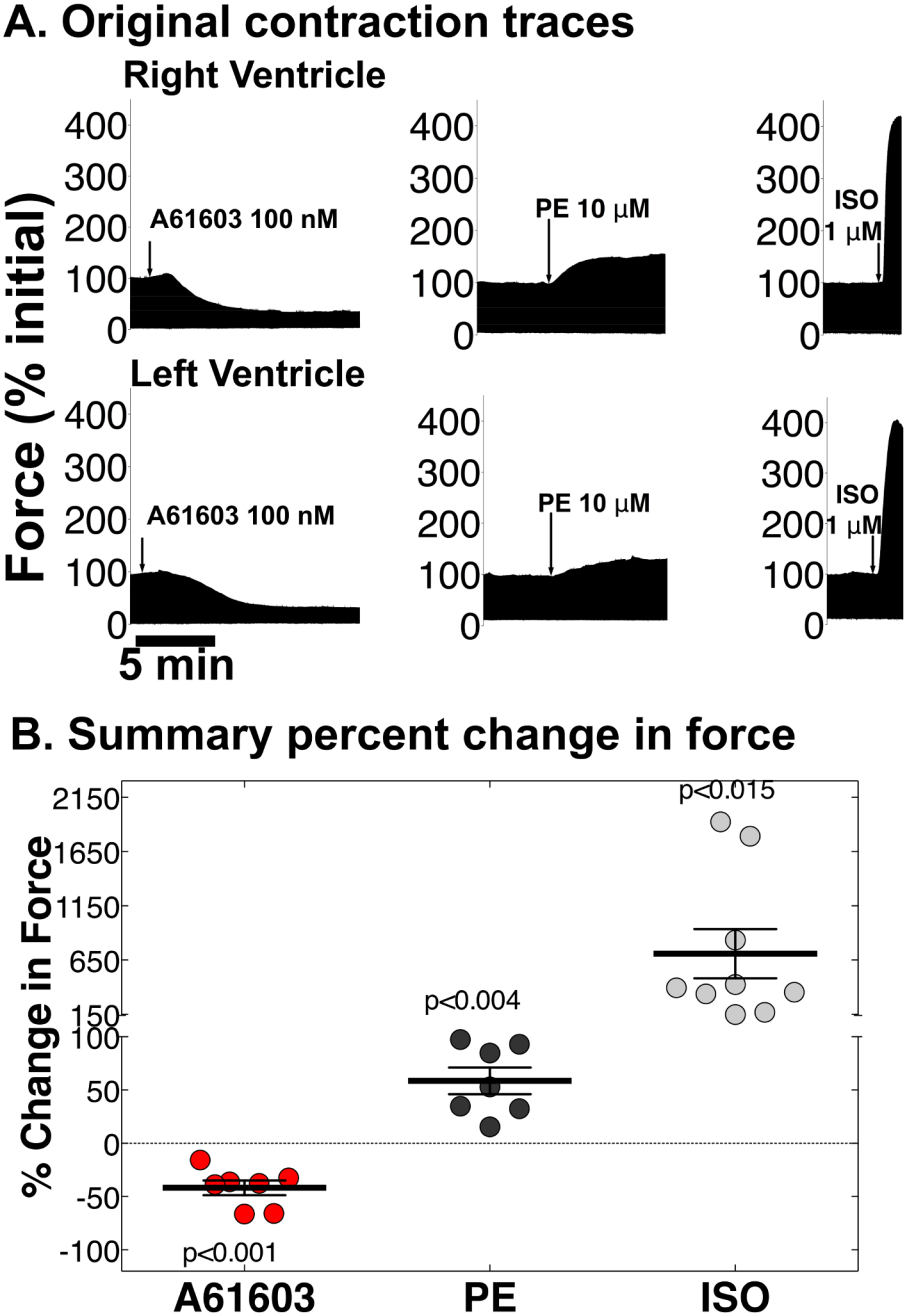
Adrenergic-mediated contraction in rabbit RV and LV. Rabbit RV and LV trabeculae were mounted, perfused, and electrically stimulated in vitro at 1.5 hz and 37°C. Contraction was measured after addition of A61603 (100 nM), the selective α1A-AR agonist; PE (10 μM), a nonselective α1-AR agonist; or ISO (1 μM), a non-selective β-AR agonist. (**A**) Original contraction traces; and (**B**) group data combining for each agonist 4–5 RV trabeculae and 3–4 LV trabeculae. Values are mean ± SE. Note split Y axis.

Fig 3A shows original contraction traces, and Fig 3B has grouped data for 7–9 hearts. A61603 at 100 nM, a maximum concentration, caused a negative inotropic effect (-42 ± 7% change in force from baseline, p<0.001). Raising A61603 to 2 μM had no effect on the negative inotropic effect of 100 nM, and did not cause a positive inotropic effect. However, subsequent addition of the nonselective α1-AR agonist PE in the same trabecula stimulated a positive inotropic effect. Overall, PE at 10 μM, a maximum dose, increased force from baseline (59 ± 12%, p<0.004). A more marked but variable positive inotropic effect was seen with the β-AR agonist ISO at 1 μM (709 ± 227%, p<0.015).

Thus these data suggested that the α1A was functional in a negative inotropic effect in rabbit myocardium. Because PE activates both the α1A and α1B, the data also suggested that the α1B mediated a positive inotropic effect.

### The α1A increases BP in the rabbit

**Fig 4:**
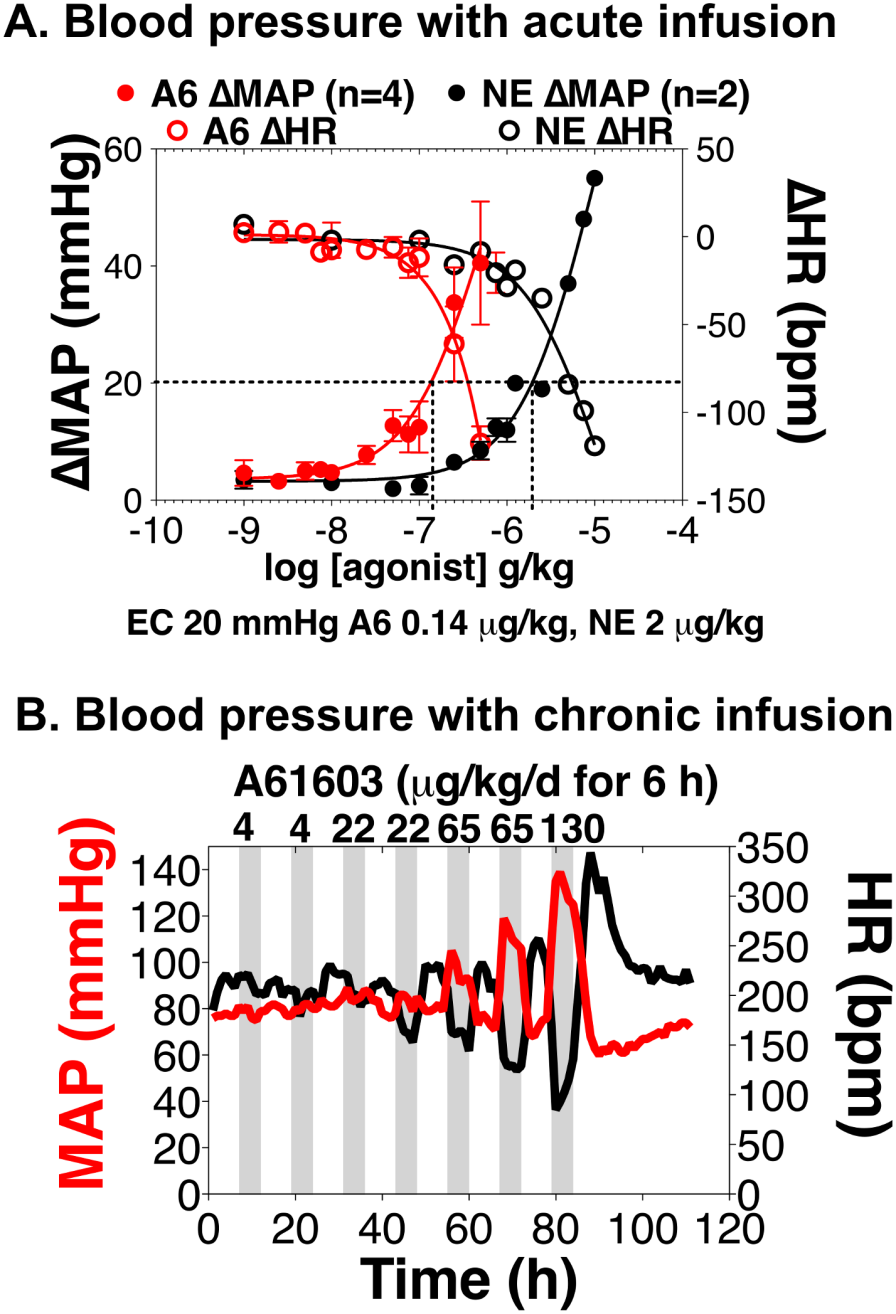
Pressor effects of A61603 in awake rabbits. BP and HR were measured in awake, restrained rabbits through a catheter in the abdominal aorta and an implanted telemetry unit. **A.** A61603 (A6) or norepinephrine (NE) was infused acutely through an ear vein catheter in restrained rabbits. Dosing that caused a 20 mmHg increase in mean arterial pressure (MAP) is indicated. **B.** A61603 was infused IV in an unrestrained rabbit through a subcutaneous iPRECIO unit, programmed to deliver the dose indicated at the top of the graph for 6 h (shaded areas), with 6 h between dosing. MAP was not changed by A61603 infused at 4 or 22 μg/kg/d.

To test vascular effects of the α1A, we measured BP in awake rabbits using a telemetry catheter in the abdominal aorta and a transmitter implanted in the flank. We used A61603 in acute and chronic IV dosing protocols. Fig 4A shows the change in mean arterial pressure (MAP) and HR with acute infusion of A61603, in comparison with the nonselective AR agonist NE. Both A61603 and NE increased MAP in a dose-related manner. The A61603 dose required to increase MAP 20 mmHg was 0.15 μg/kg, whereas the NE dose was ~10-fold higher at 2 μg/kg. HR was reduced in a reciprocal manner, reflecting the baroreflex.

Fig 4B shows MAP with chronic IV infusion of A61603. This experiment used a iPRECIO unit, programmed to deliver increasing A61603 doses for a 6 h duration at 6 h intervals. A61603 did not change MAP when delivered at 4 or 22 μg/kg/d, and increased MAP at 65 and 130 μg/kg/d. Findings were similar in a second rabbit.

### A slice model to test signaling in rabbit myocardium

We developed a slice culture model to study signaling in human myocardium, using slices 8 mm diameter and 250 μm thick [8]. We examined this model in the rabbit LV, to test α1A signaling. We made slices 5 or 8 mm diameter and 150–350 μm thick. However, despite varying multiple aspects of the human protocol in 14 rabbits, as indicated in Methods, slicing did not produce relaxed, viable slices from rabbit LV, even before calcium reintroduction.

### An anthracycline toxicity model to test cardioprotection in the rabbit

In the mouse, the α1A agonist A61603 can prevent cardiac apoptosis caused by the anthracycline doxorubicin [4]. Since anthracycline cardiotoxicity is an important clinical problem, we explored this model in the rabbit. Based on published reports, we treated 4 rabbits with daunorubicin 3 mg/kg IV weekly for 10 weeks, or 2 rabbits with doxorubicin 1 mg/kg IV weekly for 10–12 weeks [26,27,28]. Mortality was 100% before protocol completion. With daunorubicin, weight loss was 2%, versus 7% weight gain in control; echocardiographic fractional shortening, an index of LV function, fell an average 23% (range 5–40%) late in daunorubicin treatment, versus an 18% decrease in control, and troponin increased slightly, to 0.66 ng/ml versus 0.01 in control. With doxorubicin, weight loss was 12%, versus 8% weight gain in control; fractional shortening did not change, and troponin increased to 1.32 ng/ml. We concluded that the model was not a productive one and had animal welfare concerns, in our hands.

## Discussion

We quantified α1A-AR mRNA, binding, and function in the rabbit heart. The data show that α1A mRNA is a very small fraction of total α1-AR mRNA, but specific binding is detectable, at about 17% of total binding. Because total α1-AR binding is low, the absolute level of α1A-ARs is very low, <1 fmol per mg protein. Despite this low level, the α1A is functional, mediating a negative inotropic effect in rabbit myocardium in vitro. In contrast with the α1A, the α1B is predominant in rabbit heart, at mRNA and protein levels, and mediates a positive inotropic effect.

Because available commercial α1A antibodies are not valid [23], our conclusions on α1A protein and function depend on the accuracy of 2 drugs, the α1A antagonist 5MU and the α1A agonist A61603. The ability of these reagents to identify α1A-ARs is supported by studies showing loss of drug effects in α1A knockout mice [21,25].

Our data agree with reports of α1A binding in rabbit heart [12,13], but disagree with the finding of no α1A binding [10,11]. Technical differences might explain this discrepancy, such as the membrane preparation, or the antagonist used to identify the α1A in competition assays; the α1A was detected with 5MU (current study), HV723 [12], and WB-4101 [10,11], but not with KMD3213 [10,11]. We agree with the reports of very low α1A mRNA level in rabbit heart [10,11], illustrating a disconnect between mRNA and protein levels (see Table 1). We used a membrane preparation for binding that did not discard any α1-ARs.

**Table 1:** α1A-Adrenergic receptor subtype and effects in myocardium of rabbit, mouse, and man.

Element or Effect	Rabbit	Mouse	Man
α1 subtype mRNAs (%A: B: D)	0.4: 99.5: 0.1	47: 51: 2	63: 16: 21
Total α1-ARs (fmol/mg)	4	10	4
α1 subtype binding (%A: B: D)	17: 83: 0	30: 70: 0	60: 40: 0
Calculated α1A (fmol/mg)	0.8	3	2.4
A61603 LV inotropic effect	Negative	Positive	Positive
A61603 RV inotropic effect	Negative	Negative	NA
A61603 EC 20 mmHg BP (μg/kg)	0.14	0.25	NA
A61603 BP effect with chronic infusion	No effect at 22 μg/kg/d	No effect at 10 μg/kg/d	NA
Myocardial slice model	Not successful	NA	Successful
Anthracycline cardiotoxicity model	Not successful	Successful	NA

References: Rabbit: present study. Mouse: [21], [7], [4]. Man: [22],[8]. NA, not available. Note that the preparation used for radioligand binding was the same in all 3 species, and was one that did not discard any receptors, but did reduce apparent receptor density.

As in prior studies, we find that PE mediates a positive inotropic effect in rabbit myocardium [12,13,14,15]. However, those prior studies concluded that the α1A mediates a part of this positive inotropic effect, based on inhibition by presumed α1A antagonists [12,13,14,15]. Our observation that the α1A causes a negative inotropic effect is hard to reconcile with these past studies. PE activates both the α1A and α1B, so bona fide α1A antagonism would be expected to enhance a positive inotropic effect of PE, not inhibit it, by reducing the negative inotropism of α1A stimulation. Poor selectivity of available antagonists at the doses used might explain this discrepancy.

Table 1 compares α1A-AR subtype levels and effects in myocardium of rabbit, mouse, and man. Notably, α1A levels are much lower in myocardium of rabbit than the other 2 species, and the α1A inotropic effect is negative in rabbit LV, versus positive in LV of mouse and man. Given the cardiac difference in α1A function between rabbit and mouse, we tested the vascular α1A effect, and found BP regulation by A61603 in rabbit and mouse to be very similar (Table 1). Thus α1A function in rabbit LV differed from mouse, but vascular effects measured by BP did not. To test signaling and cardioprotection in rabbit, we explored a myocardial slice model useful for signaling in human myocardium [8], and an anthracycline cardiotoxicity model useful in mouse [4]. Neither of these approaches was successful in the rabbit. Prior reports used Chinchilla rabbits in an identical anthracycline model [26,27,28], versus New Zealand White in this study, and rabbit strain might be important.

## Conclusion

The α1A-AR subtype is expressed at a very low level in rabbit myocardium, but is functional, mediating a negative inotropic effect. The α1A is 3-to 4-fold more abundant in mouse and human LV myocardium, where it stimulates a positive inotropic effect. However, the effect of α1A stimulation on rabbit and mouse BP is similar. Experimental approaches useful to study signaling and cardioprotection in human myocardium or in the mouse are more challenging in the rabbit.
